# Comparing intravenous lidocaine and pethidine for pain management in emergency department patients with femoral bone fracture: a randomized controlled trial

**DOI:** 10.1186/s12871-024-02640-4

**Published:** 2024-07-23

**Authors:** Seyed Parsa Eftekhar, Ebrahim Hazrati, Reza Mosaed, Saeed Shiralizadeh Dini, Mohammad Hassan Kazemi Galougahi, Mehrshad Namazi

**Affiliations:** 1 Trauma and Surgery Research Center, AJA University of Medical Sciences, Tehran, Iran; 2 Department of Social Medicine, Faculty of Medicine, AJA University of Medical Sciences, Tehran, Iran; 3 Clinical Biomechanics and Ergonomics Research Center, AJA University of Medical Sciences, Tehran, Iran

**Keywords:** Intravenous lidocaine, Pethidine, Pain management, Emergency department, Femoral bone fracture

## Abstract

**Background:**

Intravenous lidocaine has shown promise as an effective analgesic in various clinical settings, but its utility for pain management in emergency departments, especially for bone fractures, remains relatively understudied.

**Objective:**

This study compared intravenous lidocaine to pethidine for femoral bone fracture pain management.

**Methods:**

This double-blind, randomized, controlled clinical trial was conducted in the emergency department of AJA University of Medical Sciences affiliated hospitals. Patients aged 18–70 years-old with femoral bone fracture and experiencing severe pain, defined as a numerical rating scale (NRS) of pain ≥ 7, were included in the study. One group received intravenous pethidine (25 mg), while the other group received intravenous lidocaine (3 mg/kg, not exceeding 200 mg), infused with 250 ml saline over 20 min. Pain levels were evaluated before treatment administration (0 min) and at 10, 20, 30, 40, 50, and 60 min after treatment administration using the NRS.

**Results:**

Seventy-two patients were enrolled in the study. Demographic characteristics and pain scores were similar between the two groups. The mean pain scores upon arrival for the lidocaine and pethidine groups were 8.50 ± 1 and 8.0 ± 1, respectively; after one hour, they were 4.0 ± 1 and 4.0 ± 1, respectively. While there was a statistically significant reduction in pain in both groups after one hour, there were no clinically or statistically significant differences between the two groups (*p* = 0.262). Pethidine had a higher incidence of adverse events, though not statistically significant. Additionally, females required more rescue analgesics.

**Conclusion:**

The administration of intravenous lidocaine is beneficial for managing pain in femoral bone fractures, suggesting that lidocaine could be a potent alternative to opioids.

**Trial Registration:**

IRCT20231213060355N1 (https://irct.behdasht.gov.ir/trial/74624) (30/12/2023).

## Introduction

Bone fractures are a prevalent reason for seeking emergency care, with nearly 2 million individuals admitted to emergency departments in the United States annually due to long bone fractures [1]. The incidence of femoral shaft fractures ranges from 9.5 to 18.9 per 100,000 persons-year [2], often resulting in moderate to severe pain among affected patients. Despite the high prevalence of fractures and associated pain, initial pain management in the emergency department is frequently suboptimal, with over 50% of patients expressing dissatisfaction with their pain management strategies [3]. Clinicians commonly prescribe a variety of analgesics to alleviate fracture-related pain, depending on the severity of injury and individual patient factors. Intravenous opioids such as morphine, pethidine, and hydrocodone are frequently utilized as the primary agents for pain management in patients with moderate to severe isolated limb trauma, while non-opioid oral medications like non-steroidal anti-inflammatory drugs (NSAIDs) and paracetamol are typically reserved for less severe injuries [3].

Lidocaine, a widely employed amide local anesthetic, exerts its analgesic effects by blocking sodium channels in peripheral and central neurons of the nociceptive pathway [4]. Although primarily used for nerve blocks and infiltration anesthesia, intravenous lidocaine has demonstrated efficacy as an analgesic agent when administered systemically [5]. Randomized clinical trials conducted in surgical settings have reported that intravenous lidocaine reduces pain intensity, diminishes postoperative opioid consumption, and shortens hospital stays among surgical patients [6, 7]. Additionally, intravenous lidocaine has shown promise in managing refractory cancer pain and neuropathic pain [8, 9].

However, the utility of intravenous lidocaine for pain management in the emergency department setting remains relatively understudied. While the American College of Emergency Physicians advocates for the preferential use of non-opioid medications as first-line treatment for fracture-related pain [10], emerging evidence suggests that intravenous lidocaine may offer effective pain relief in acute conditions such as renal colic and critical limb ischemia, potentially surpassing the potency of morphine [11, 12]. Nonetheless, robust evidence supporting the superiority of intravenous lidocaine over opioids for bone fractures in the emergency department is lacking. In light of this gap in the literature, our study aims to investigate the safety and efficacy of intravenous lidocaine compared to pethidine for pain control in patients presenting with femoral bone fractures in the emergency department.

## Methods

### Design and setting

This double-blind, randomized, controlled clinical trial was conducted at AJA University of Medical Sciences affiliated hospitals, including Besat, Imam Reza, Khanevade, and Golestan hospitals, located in Tehran, Iran, from January 2024 to April 2024. The study protocol received approval from the Ethics Committee of AJA University of Medical Sciences (Ethical code: IR.AJAUMS.REC.1402.168) and was registered with the Iranian Registry of Clinical Trials (IRCT ID: IRCT20231213060355N1; registration date: 30/12/2023). Prior to enrollment, written informed consent was obtained from all study participants.

### Participants

Patients admitted to the emergency department of AJA University Hospitals with a diagnosis of traumatic femoral bone fracture underwent screening for eligibility based on predetermined criteria. Inclusion criteria encompassed individuals aged between 18 and 70 years, confirmed femoral bone fracture via x-ray examination, and reported severe pain, defined as a NRS score of 7 or higher. Exclusion criteria comprised crush injuries of limbs and open fractures, acute diseases other than fractures, pregnancy, history or presence of cardiac block or bradycardia, history or presence of seizure, neuromuscular diseases, neuropathic diseases, diabetes, history of analgesic or anti-inflammatory consumption, history of opioid consumption, and documented allergic reactions to lidocaine and pethidine. The patient selection process was conducted by emergency medicine physicians.

### Randomization

A computer-generated permuted block randomization list was prepared by an independent statistician with no clinical involvement in the study. This list was generated using a validated randomization algorithm to ensure randomness and unpredictability in the assignment of participants to treatment groups. The randomization list was structured in blocks, with each block containing four participants. This block size was chosen to minimize predictability in group assignment while maintaining balance between the treatment and control groups. The allocation ratio was set at 1:1, ensuring equal distribution of participants between the treatment and control groups. Participant allocation information was concealed within sealed envelopes, preventing researchers and participants from accessing the randomization sequence during the study. This maintained the integrity of the blinding process and minimized selection bias. Upon enrollment, participants were sequentially assigned to treatment groups based on their order of entry into the study. Each participant was allocated to the next available slot in the randomization sequence according to the permuted block design. Both participants and researchers involved in the study were blinded to the treatment assignment throughout the trial. The treatments were prepared by a nurse. Participants received identical-looking treatments, and researchers were provided with coded treatment labels to maintain blinding during data collection and analysis. Adherence to the randomization protocol was closely monitored throughout the study to ensure the integrity of the blinding process. Any deviations from the protocol were documented and addressed promptly to maintain the validity of the study results.

### Intervention

Patients admitted to the emergency department with femoral fractures confirmed by x-rays were included in the study. In addition to standard fracture management procedures, such as limb immobilization with a temporary splint, patients were informed about the purpose and methodology of the study by the emergency physician. Eligibility for participation was assessed, and written informed consent was obtained from eligible patients.

Two sets of sterile, colorless, and ready-to-inject 10 milliliters (ml) syringes were prepared and labeled as A and B. Syringe A contained 0.5 ml of pethidine (50 mg/ml) diluted with 9.5 ml of sterile water for injection, resulting in a total volume of 10 ml and a pethidine concentration of 25 mg. Syringe B contained 10 ml of lidocaine 2% (20 mg/ml), resulting in a total lidocaine dose of 200 mg. The dosage of pethidine was fixed at 25 mg, while the dosage of lidocaine was calculated at 3 mg/kg (not exceeding 200 mg to prevent toxicity) [13, 14].

During the study, the prepared treatments were administered intravenously over a period of 20 min using 250 ml of normal saline solution. Administration of the treatments was conducted under the supervision of complete cardiac and respiratory monitoring to ensure patient safety and proper treatment infusion.

### Outcomes

The primary outcome of this study was the assessment of pain severity based on the NRS, with scores ranging from 0 (indicating no pain) to 10 (representing the worst pain imaginable). Pain intensity was evaluated at various time points: before treatment administration (0 min), as well as 10, 20, 30, 40, 50, and 60 min after treatment administration [15]. This allowed for a comprehensive understanding of the treatment’s effect on pain relief over time.

The secondary outcome focused on identifying and monitoring adverse events associated with the administered treatment. Adverse events included seizure, cardiac arrhythmia, headache, nausea, dry mouth, and any other signs or symptoms reported by the patients. The occurrence of adverse events was systematically recorded to assess the safety profile of the treatment. In cases where pain persisted after 30 min following treatment administration and the reduction in pain was less than 30%, or upon patient request for analgesics, intravenous fentanyl (1.5 µg/kg) was administered as a rescue dose. This intervention aimed to provide additional pain relief to patients experiencing inadequate pain control with the initial treatment regimen [12, 15].

Throughout the study duration, patients’ vital signs, including heart rate, respiratory rate, blood pressure, and temperature, were closely monitored. Vital sign monitoring was conducted before, during, and after treatment administration to ensure patient safety and to promptly identify any adverse physiological responses associated with the treatment.

### Sample size

The sample size for this study was determined using power analysis with a significance level (α) of 0.05 and a desired statistical power of 80%. The expected effect size was estimated at 0.7 based on previous research [16]. Given these parameters, the sample size required for each group was calculated using a standard formula for comparing means in two independent groups. Utilizing statistical software G*Power, the sample size calculation yielded 33 participants per group. To account for potential dropouts or non-compliance, the final sample size was increased by 10%, resulting in a total target sample size of 72.

### Statistical analysis

Descriptive statistics, including mean ± standard deviation (SD) for normally distributed quantitative variables and median with interquartile range (IQR) for non-normally distributed quantitative variables, were utilized to summarize baseline characteristics such as age, weight, height, and body mass index (BMI). To compare baseline characteristics between the treatment groups, independent t-tests were employed for continuous variables. The Wilcoxon signed-rank test was used to assess changes in pain severity within each treatment group before and after treatment administration. Additionally, the Mann-Whitney U test was utilized to compare pain severity and pain reduction between the two treatment groups at different time points. For qualitative variables, such as treatment adverse events, Fisher’s exact test and chi-squared test were employed to compare proportions between the treatment groups. Logistic regression analysis was performed to identify determinants associated with pain recurrence and the use of fentanyl as rescue analgesia.

## Results

**Fig. 1:**
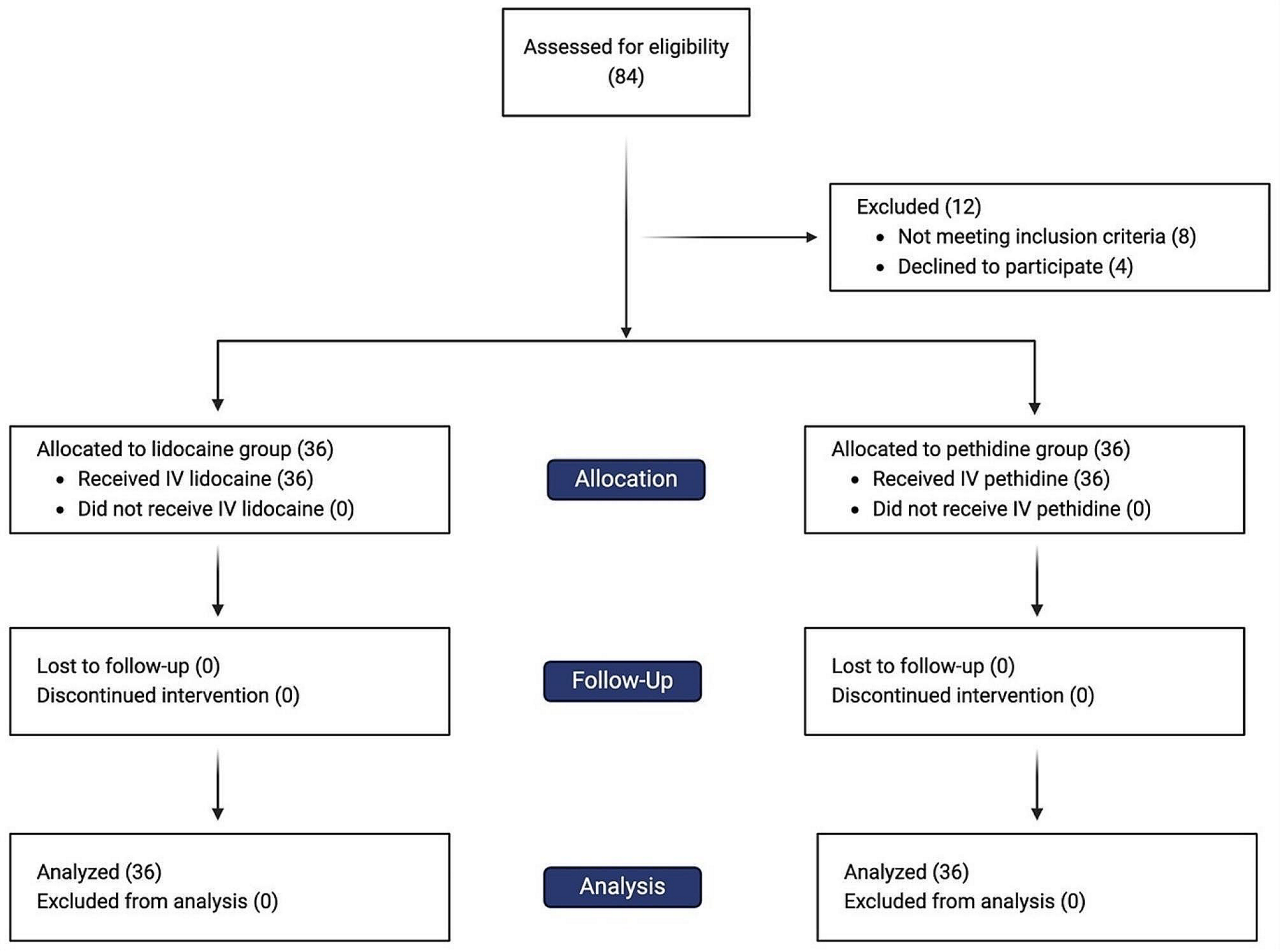
CONSORT diagram

During the study period, spanning from January 1 to April 1, 2024, a total of 84 patients were screened for eligibility, out of which 72 patients were enrolled and randomized. Figure 1 shows the CONSORT flow chart of study patients. Complete data on primary and secondary outcomes were available for all enrolled patients. Patient demographics are summarized in Table 1. No statistically significant differences were observed in gender distribution, weight, height, and BMI between the two treatment groups.

**Table 1:** Patients’ demographics characteristics

Characteristics	Pethidine *N* (%)	Lidocaine *N* (%)	*p*-value
Gender			
Male	19 (52.77)	22 (61.11)	0.344
Female	17 (47.23)	14 (38.89)	
Age (years), mean ± SD	34.6 ± 9.8	34.2 ± 7.9	0.823
BMI (kg/m2), mean ± SD	25.6 ± 3.1	26.3 ± 2.7	0.314

**Fig. 2:**
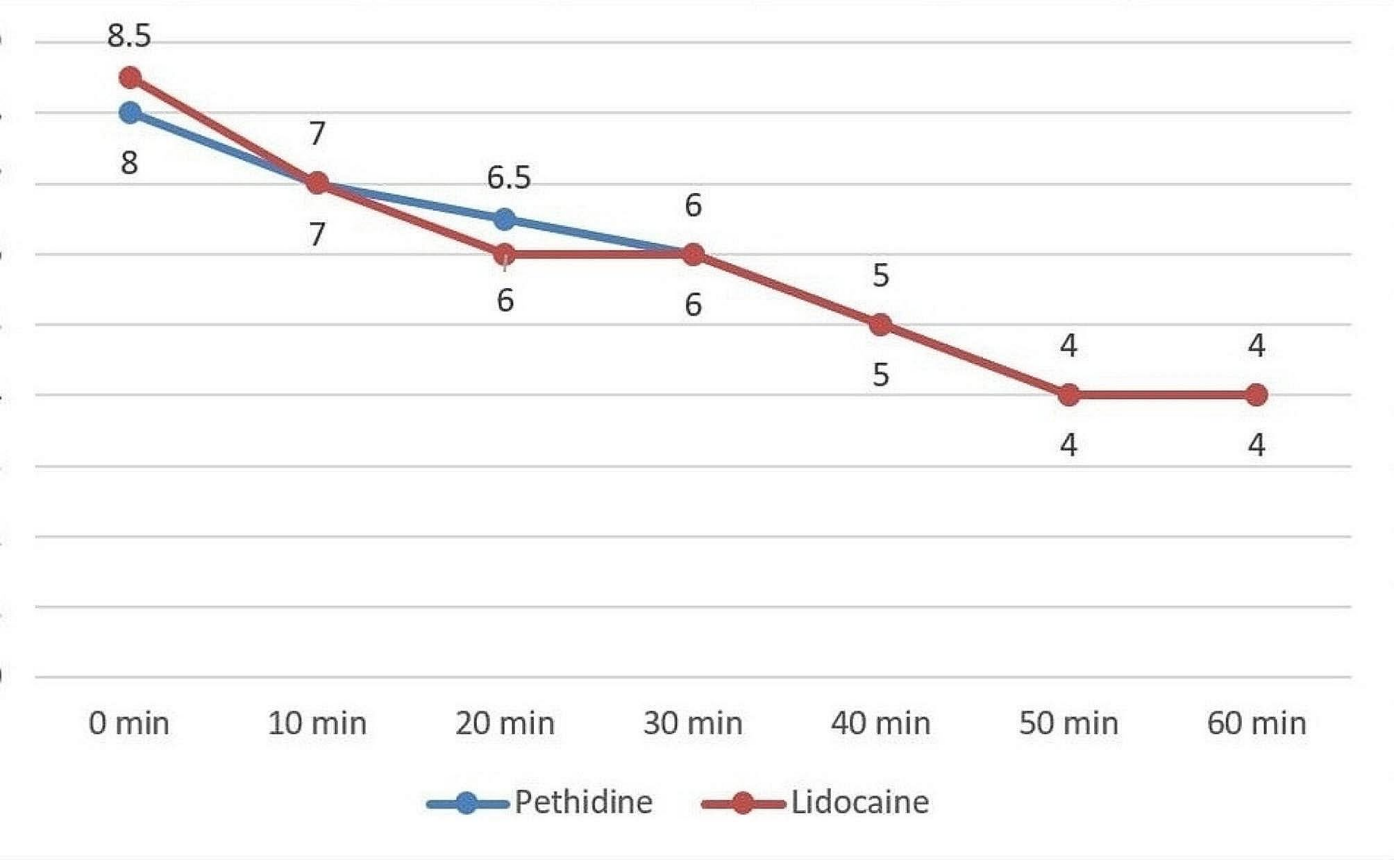
Comparison of pain severity, as assessed by the numerical rating scale (NRS), across various time points between participants administered with pethidine and lidocaine. This plot illustrates the dynamic changes in pain perception over time for both treatment groups, shedding light on the efficacy of pethidine and lidocaine in managing pain

Analysis using the Wilcoxon signed-rank test revealed a significant reduction in pain severity within both the pethidine and lidocaine groups after 10 min of treatment administration (pethidine group: Z = -5.219, *p* < 0.001; lidocaine group: Z = -5.015, *p* < 0.001). However, there were no significant differences in pain severity (NRS) between the two groups at various time points, including at the time of treatment administration and at 10, 20, 30, 40, 50, and 60 min post-administration (Table 2; Fig. 2).

**Table 2:** Pain severity in different time points (before and after treatment administration) based on numerical rating scale (NRS).

Time	Pethidine Median ± IQR	Lidocaine Median ± IQR	*p*-value
Before administration (0 min)	8.0 ± 1	8.50 ± 1	0.939
10 min	7.0 ± 2	7.0 ± 2	0.433
20 min	6.50 ± 1	6.0 ± 1.75	0.742
30 min	6.0 ± 1	6.0 ± 2	0.537
40 min	5.0 ± 1	5.0 ± 1.75	0.494
50 min	4.0 ± 1	4.0 ± 1	0.383
60 min	4.0 ± 1	4.0 ± 2	0.634

Rescue analgesia (fentanyl) was administered to 36.1% of patients in the pethidine group and 25% of patients in the lidocaine group after 30 min of initial treatment administration, with no statistically significant difference observed between the two groups (*p* = 0.306). Notably, a higher proportion of females (48.4%) compared to males (17.1%) received fentanyl as rescue analgesia (*p* = 0.005). Further analysis adjusting for gender and age groups revealed that female gender was independently associated with a higher likelihood of receiving fentanyl as rescue analgesia (OR: 4.64; 95% CI: 1.534–14.054; *p* = 0.007) (Table 3).

**Table 3:** Evaluating the determinants associated with pain recurrence and the use of fentanyl as rescue analgesia. Logistic regression was used to calculate the adjusted odds ratio (OR) for different age groups and gender

Characteristics	Coefficient β	OR (95% CI)	*p*-value
Age (years)			
20–29 (Reference)	–	–	–
30–39	− 0.214	0.807 (0.221–2.945)	0.746
40–49	0.513	1.671 (0.369–7.556)	0.505
≥ 50	1.242	3.464 (0.410–29.253)	0.254
Gender			
Male (Reference)	–	–	–
Female	1.535	4.643 (1.534–14.054)	0.007*

*p-value ≤ 0.05

Although the prevalence of treatment adverse events was higher in the pethidine group compared to the lidocaine group (25% vs. 13.9%, *p* = 0.23), this difference was not statistically significant. Specifically, the incidence of headache and nausea did not significantly differ between the two groups, with rates of 8.3% and 11.1% for headache and 5.6% and 8.3% for nausea in the pethidine and lidocaine groups, respectively (*p* = 1.000). Additionally, while the incidence of dry mouth was higher in the pethidine group (two patients) compared to the lidocaine group (zero patients), this difference was not statistically significant (*p* = 0.493).

## Discussion

This study demonstrates that both pethidine and lidocaine significantly reduce acute pain severity in patients with femoral bone fractures. There was no significant difference in pain severity at different time points after treatment administration between the two groups. Additionally, the occurrence of treatment adverse events was similar between the two groups. Furthermore, our findings suggest that female gender is associated with an increased need for rescue analgesic.

Opioids such as morphine, pethidine, oxycodone, and hydrocodone are the mainstay of acute pain management in the emergency department. However, the growing misuse of opioids is a significant concern [17]. Previous research has indicated that intravenous lidocaine may be beneficial in managing acute pain in patients with renal colic and critical limb ischemia, potentially even more potent than morphine [11, 12]. Our study demonstrates that continuous intravenous lidocaine administration (within 20 min) significantly reduces pain in femoral bone fractures, with analgesic properties similar to intravenous pethidine. Lidocaine has been shown to possess anti-hyperalgesic, analgesic, and anti-nociceptive effects [18]. However, the precise analgesic mechanism of intravenous lidocaine remains incompletely understood. Several mechanisms may play a role, such as the inhibition of potassium, sodium, and calcium channels, glutamate receptors, and G-protein-coupled receptors [19]. Moreover, intravenous lidocaine may involve the inhibition of transient receptor potential channels, hyperpolarization-activated cyclic nucleotide-gated channels, acid-sensing ion channels, acetylcholine receptors, serotonin receptors, among others [20]. It appears that the analgesic effect of intravenous lidocaine is a multifactorial phenomenon, and no definitive single molecular mechanism has been identified.

Farahmand et al. evaluated the efficacy of intravenous lidocaine (1.5 mg/kg) in 50 patients with extremity trauma in the emergency department with a pain score (NRS) higher than 4. They found that lidocaine can be an effective analgesic in traumatic patients, with its analgesic effect similar to intravenous morphine (0.1 mg/kg) [16]. Additionally, Forouzan et al. investigated the efficacy of intravenous lidocaine (1.5 mg/kg) in patients with extremity fractures and found that it could significantly reduce pain in these patients. However, they did not specify the type of fracture [21]. Our findings were consistent with the mentioned studies; however, we utilized a larger sample size, which increases the power of our study, and we specified the type of fracture (femoral bone fracture).

In our study, the incidence of treatment adverse events, including nausea, headache, and dry mouth, was higher in the pethidine group compared to the lidocaine group. However, this difference was not statistically significant. Although not statistically significant, minor differences could be clinically relevant, especially in patients with a history of adverse drug events due to opioids. We excluded patients with a history of cardiac block, arrhythmia, and seizures. Intravenous lidocaine should be cautiously administered due to its cardiotoxicity and neurotoxicity, especially in patients with comorbidities such as cardiac failure, heart block, and epilepsy. Moreover, further studies are necessary to assess the safety of intravenous lidocaine in patients with comorbidities.

The narrow therapeutic index of lidocaine necessitates constant cardiac and respiratory monitoring of patients, as we monitored all patients during treatment administration. Initial manifestations of lidocaine toxicity include numbness of the tongue, metallic taste, tinnitus, and drowsiness, while higher doses may lead to visual disturbances, muscle twitching, and seizures [22]. However, the incidence of lidocaine-associated adverse events appears to be largely dose-dependent [23]. In our study, we did not observe these adverse events, suggesting that continuous administration of intravenous lidocaine may help prevent treatment-related adverse events.

The need for rescue analgesics, a surrogate marker for the amount of pain experienced by patients, is crucial for evaluating the efficacy of an analgesic in any trial [23]. We found that the demand for rescue analgesic fentanyl (1.5 µg/kg) was higher in the pethidine group; however, this difference was not statistically significant. Moreover, female gender was associated with an increased likelihood of requiring rescue analgesic. Farahmand et al. reported that the demand for recue analgesic was similar in patients with extremities trauma in both morphine and lidocaine group [16]. Zhong et al. evaluated the demand for rescue analgesic in various emergency department situations and found a nonsignificant higher demand for rescue analgesic in the intravenous lidocaine group compared to the opioid group. However, due to limited studies reporting the need for rescue analgesic, they noted in their meta-analysis that this finding is not definitive [23]. Furthermore, studies have revealed that women are more sensitive to pain compared to men [24]. Although not yet influencing clinical practice, investigating sex differences in pain may have important implications for the development of new analgesics.

Our study had several limitations. We excluded patients under 18 years old and over 70 years old, as well as those with a history of cardiac arrhythmias and seizures. Further studies are warranted to evaluate the efficacy of intravenous lidocaine in these populations. We limited the maximum dose of intravenous lidocaine to 200 mg to prevent toxicity; however, this can result in a suboptimal dosage for some patients. One of the limitations of our study is that the medication was prepared by nurses. Although the nurses were trained, this could still introduce bias. The majority of studies have investigated the efficacy of intravenous lidocaine in a surgical setting [25]. Furthermore, the literature is limited regarding the administration of intravenous lidocaine in emergency situations, particularly for bone fractures. Our study is the first to specify the type of fracture (femoral bone fracture). We utilized a larger sample size compared to previous studies, increasing the robustness of our findings. Moreover, ours is the first study to investigate the efficacy of continuous administration of intravenous lidocaine in acute pain management in the emergency department (within 20 min), allowing us to use a higher dosage of lidocaine compared to previous studies.

## Conclusions

In conclusion, the administration of continuous intravenous lidocaine demonstrated beneficial effects in alleviating the pain of femoral bone fractures in the emergency department. Furthermore, intravenous lidocaine can serve as a safe alternative to pethidine and may help reduce the misuse of opioids.

## Data Availability

The data supporting the findings of this study are available on request from the corresponding author and with permission from the AJA University of Medical Sciences, Tehran, Iran.

## Abbreviations

NRS: Numerical rating scale
NSAID: Non-steroidal anti-inflammatory drug
SD: Standard deviation
IQR: Interquartile range
BMI: Body mass index
